# Construction of CeRNA regulatory network based on WGCNA reveals diagnosis biomarkers for colorectal cancer

**DOI:** 10.1186/s12885-022-10054-z

**Published:** 2022-09-17

**Authors:** Jun Xiang, Li Gao, Hao Yu Jing, Yun Xiao Liu, Hu Fei Wang, Ze Wen Chang, Shi Hao Liu, Lei Yu, Gui Yu Wang

**Affiliations:** 1 Department of Colorectal Surgery, The Second Affiliated Hospital of Harbin Medical University, Harbin, Heilongjiang People’s Republic of China; 2 Department of Oral and Maxillofacial Surgery, The Second Affiliated Hospital of Harbin Medical University, Harbin, China

**Keywords:** ceRNA, Colorectal cancer, WGCNA, Network, Targets, TCGA

## Abstract

**Background:**

Colorectal cancer is the third most common cause of death among cancers in the world. Although improvements in various treatments have greatly improved the survival time of colorectal cancer patients, since colorectal cancer is often at an advanced stage when diagnosed, the prognosis of patients is still very poor. Since the ceRNA regulatory network was proposed in 2011, it has greatly promoted the study of the molecular mechanism of colorectal cancer occurrence and development.

**Objective:**

Exploring the new molecular mechanism of colorectal cancer occurrence and development and providing new targets for the diagnosis and treatment of colorectal cancer.

**Method:**

We analyzed the RNA-seq data of CRC from TCGA, such as differential expression analysis, weighted gene co-expression network analysis (WGCNA) and construction of ceRNA regulatory network.

**Results:**

We constructed a ceRNA network using RNA-seq data of CRC from TCGA. In the ceRNA regulatory network, 19 hub molecules with significant prognostic effects were ultimately identified, including 8 lncRNAs, 2 mRNAs and 9 miRNAs. These hub molecules constitute the lncRNA-miRNA, miRNA-mRNA or lncRNA-miRNA-mRNA axis.

**Conclusion:**

In this article, some new ceRNA regulatory axes have been discovered, which may potentially disclose new molecular mechanisms for the occurrence and development of colorectal cancer, thereby providing an important blueprint for the treatment and prognosis assessment of CRC patients.

**Supplementary Information:**

The online version contains supplementary material available at 10.1186/s12885-022-10054-z.

## Introduction

Colorectal cancer (CRC), one of the most common malignant tumors in the world, is the third leading cause of cancer deaths worldwide and causes about 700,000 deaths per year [1, 2]. According to a recent report by the International Agency for Cancer Research, CRC accounts for 6.3% of all cancer deaths in China [3]. Due to the lack of specific and sensitive biomarkers, CRC patients are usually diagnosed as advanced cancer and the overall five-year survival rate is 40 to 60% [4, 5]. Although the progress of interventional therapy has considerably improved the overall survival (OS) of CRC in recent years, the prognosis of patients is still inferior due to the high recurrence and metastasis rate of advanced CRC [6]. As a multistage disease, CRC is the accumulation of multiple genetic or epigenetic changes and their complex mutual effects [7]. In order to improve the early diagnosis, treatment and prognosis of CRC, it is necessary to study the molecular mechanism of CRC from initiation to metastasis. In recent years, there have been countless reports of lncRNA regulating the different biological behaviors of CRC cells via the ceRNA regulation network. For example, lncRNA CCAT1 functions as ceRNA to antagonize miR-401 in down-regulating ITPKB in human colon cancer cell HCT116 [8]. In addition, it has been reported that lncRNA CLM regulates the expression of ZEB1 as a ceRNA of miR-215 to promote liver metastasis of CRC [9]. In addition, the newly discovered lncRNA TUSC7 inhibits the proliferation of CRC cells through the molecular sponge miR-211 [10].

Non-coding RNA (ncRNA) refers to transcripts without protein coding function, and its number accounts for more than 98% of the entire genome transcript [11]. The definition of long non-coding RNA (lncRNA) is RNA transcripts that are more than 200 nucleotides in length and cannot encode proteins. Although most lncRNAs have poly-A tails, they cannot be translated into proteins. Compared with protein-encoding mRNA, lncRNA shows greater tissue specificity, so it may become a biomarker for many diseases [12]. MicroRNAs (miRNAs), non-coding RNAs with a length of 19-22 bases, were first detected in eukaryotes, which can regulate endogenous genes. MiRNAs degrade messenger RNAs (mRNAs) or inhibit protein translation to regulate them at the posttranscriptional gene expression level [13, 14]. LncRNA uses various mechanisms to regulate the expression, degradation, and modification of proteins. The most vital regulation is the competitive endogenous RNA (ceRNAs) theory proposed by Salmena et al. [15]. The ceRNA hypothesis describes the intricate posttranscriptional communication network of all transcripts, including lncRNA RNA species, which can be used as natural miRNA sponges to inhibit miRNA function by sharing miRNA response elements (MRE) [16]. The importance of the lncRNA-miRNA-mRNA regulatory network in different diseases was confirmed by subsequent studies [17].

In the past few decades, only a small number of lncRNAs have been well identified for their biological functions in the occurrence and development of CRC. In this study, we analyzed the RNA-seq data of CRC in TCGA to determine lncRNAs, miRNAs and mRNAs that are significantly changed during the development of CRC. Subsequently, we constructed the ceRNA regulatory network of these lncRNAs, miRNAs and mRNAs to clarify the molecular mechanism of CRC occurrence and development, and to find a new way to predict the occurrence and development of CRC, so as to provide new ideas for clinical diagnosis and therapy.

## Materials and methods

### Data download and processing

The data used in this research are from online public databases, downloading CRC data from the GEO database. The inclusion criteria are: 1. There are at least 10 tumor and normal tissue samples; 2. The data includes tumor and normal samples; 3. The sample data has not been processed. Finally, GEO data was obtained, including GSE156355, GSE110224, GSE110223, GSE41657, GSE113512, GSE50117, GSE103512. In addition, the count data of CRC and related clinical information data were downloaded from the TCGA database. A total of 697 RNA-seq data were downloaded. It primarily includes 17,580 mRNAs, 7365 lncRNAs and 802 miRNAs data.

The data obtained from GEO uses the R package limma (version 3.48.3) for correction and normalization, and log2 conversion is performed on the data. The CRC data obtained in TCGA uses the ENSEMBL database for gene annotation. In the meantime, the average value of the gene, which has a repetition name, is taken and the genes that are less than 30% expressed in the sample are finally removed.

### Screening of differentially expressed LncRNA and mRNA

The R package DESeq2 (version 1.32.0) to analyze the differentially expression of the TCGA count data. For the *p*-value, we use false discovery rate (FDR) to correct for the statistical significance of multiple tests. The final result uses |log2FC| > 1 and FDR < 0.05 as cutoff criteria to screen differentially expressed genes (DEGs) and differentially expressed lncRNAs (DELs).

### Weighted gene co-expression network analysis

The differentially expressed genes screened in the TCGA CRC data were selected to establish a weighted gene co-expression network. After selecting the appropriate samples and genes, using the WGCNA (version 1.70-3) R package to calculate the “Pearson correlations coefficient” between all gene pairs in the selected samples to construct an adjacency matrix. Then, using the soft-threshold power (β) as 6 to construct a scale-free network (scale-free R2 = 0.90). In order to further recognize the functional modules in the co-expression network with these 5201 genes, the adjacency matrix is used to calculate the Topological Overlap Measure (TOM), which represents the overlap in the shared neighbors.

Modular engines (MEs) are considered to be representative of gene expression profiles in the modules. Select modules related to tumor function for subsequent analysis.

### GO enrichment analysis and KEGG pathway analysis

Performing GO enrichment and KEGG pathway analysis on the characteristic genes of different modules by the R package clusterProfiler (version 4.0.5) [18–20]. We select appropriate modules for further analysis according to the GO enrichment and KEGG pathway analysis results of each module. *P*-value< 0.05 is considered to be a significant enrichment analysis result.

### Construction of ceRNA network and topology analysis

After predicting miRNAs in the miRcode and ENCORI databases, we obtained miRNAs that interact with various lncRNAs, and overlapped these miRNAs with miRNAs in TCGA CRC to obtain the final lncRNA-miRNA relationship file. We used miRTarBase and miRWalk databases to predict the target genes of these miRNAs, and overlapped the genes predicted by the two databases with the eigengenes genes of the module, and obtained miRNA-mRNA relationship pairs.

We merged the gained lncRNA-miRNA and miRNA-mRNA two relationships pairs and applied them to the Cytoscape software to visualize the topological network to obtain the topological network diagram of ceRNA. Performing topology analysis on the network, hub nodes were selected with a degree greater than 5 to construct a subnetwork and perform subsequent analysis.

### Statistical analysis

Using the analysis tool R x64 4.0.4 and the online analysis tool “Xiantao Academic” (https://www.xiantao.love/) to perform statistical analysis. It mainly includes differentially expression analysis, ROC analysis, and survival analysis. In survival analysis, the Kaplan-Meier method and the log-rank test were used. *P*-value < 0.05 was considered statistically significant.

## Results

**Fig. 1:**
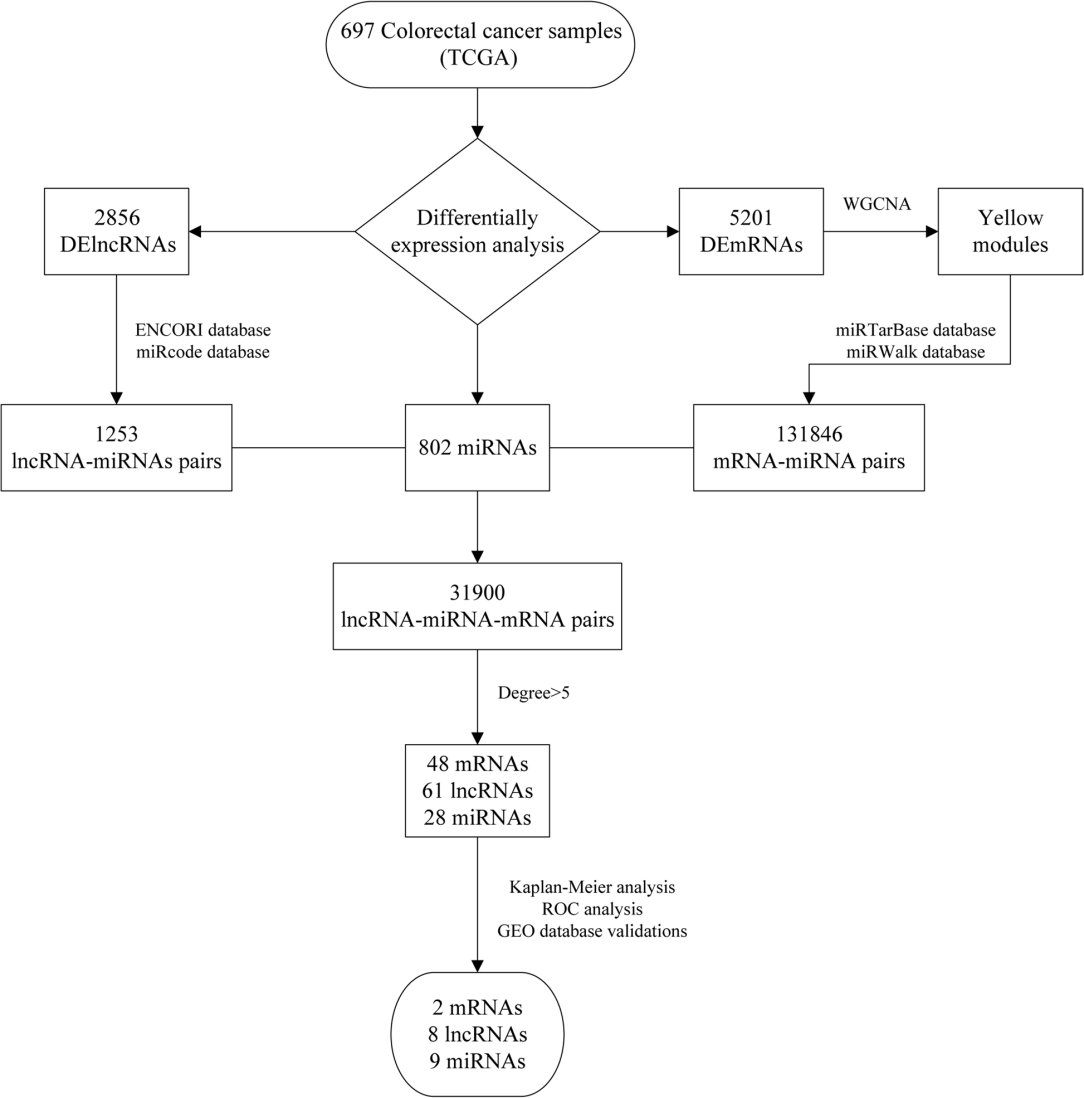
Working diagram of this study

The workflow is shown in Fig. 1.

### Determination of significantly differentially expressed mRNA and significantly differentially expressed lncRNA

**Fig. 2:**
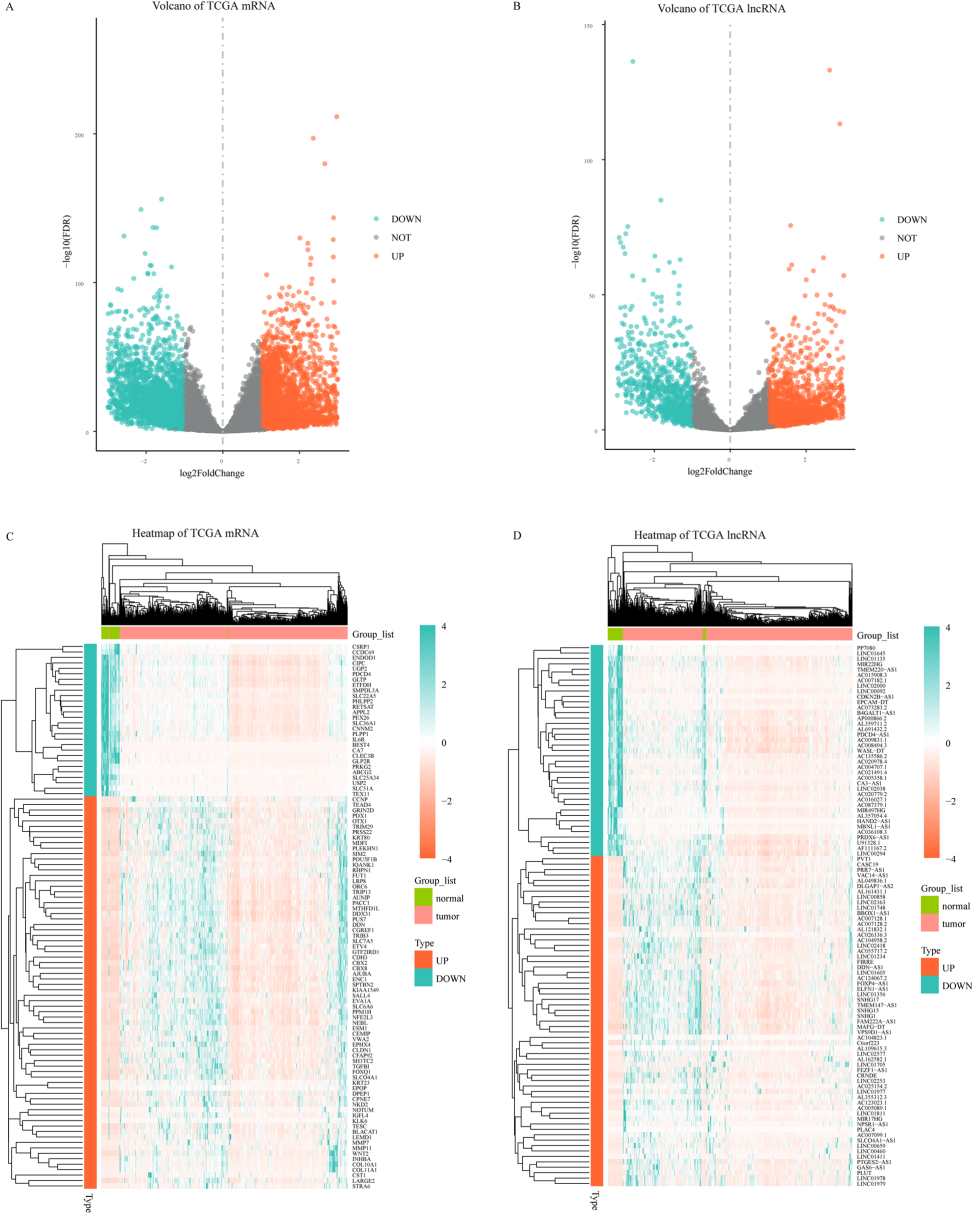
Differential expression analysis of mRNA and lncRNA in TCGA colorectal cancer. **A** shows the volcano map of mRNA, **B** shows the volcano map of lncRNA, **C** shows the clustering heat map of differentially expressed mRNA, **D** shows the clustering heat map of differentially expressed lncRNA. In **C** and **D**, “Group_list” represents whether the sample is a normal sample or a tumor sample, “Type” represents the differential expression type of each gene (significantly up-regulated or significantly down-regulated)

The mRNA expression profiles of 51 normal samples and 646 tumor samples were compared. A total of 5201 differentially expressed mRNAs were obtained through statistical testing, including 2653 up-regulated and 2548 down-regulated (Fig. 2A and C).

The lncRNA expression profiles of 51 normal samples and 646 tumor samples were compared. A total of 2856 differentially expressed lncRNAs were obtained through statistical testing, including 1981 up-regulated and 875 down-regulated (Fig. 2B and D).

### Weighted gene co-expression network analysis results

**Fig. 3:**
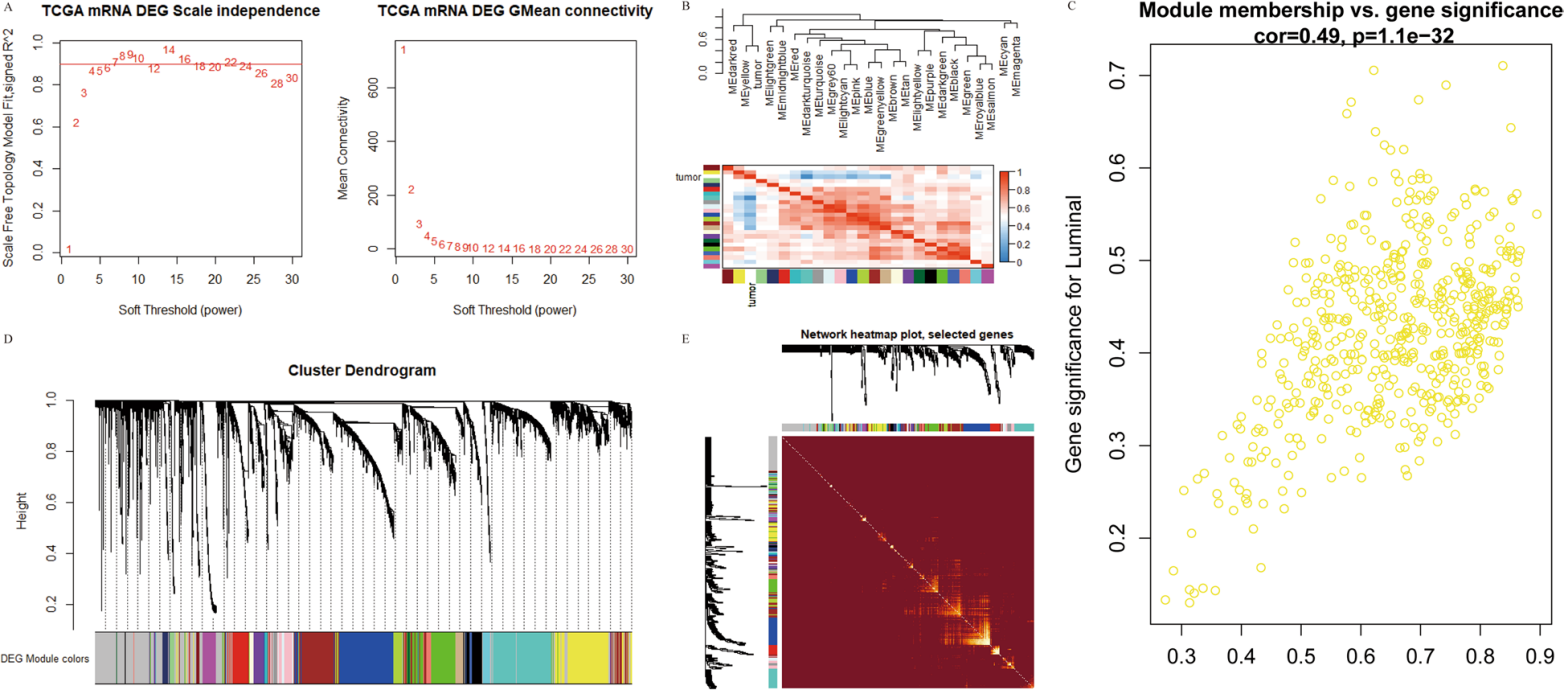
Construction of WGCNA. **A** shows the scale-free fitting index of the network topology obtained by the soft-threshold power analysis method. **B** shows the connectivity of characteristic genes. Red indicates positive correlation and blue indicates negative correlation. **C** is the analysis of gene significance and module membership in the key module yellow. **D** level cluster analysis is used to detect co-expression clusters with corresponding color assignments. Each color represents a module in the gene co-expression network constructed by WGCNA. **E** is to visualize some random genes from the network, using a heat map to describe the TOM between genes in the analysis. On a linear scale, the depth of red is positively correlated with the correlation strength between the pair of modules

The aforementioned significantly differentially expressed mRNA was used to construct WGCNA, and a total of 5201 significantly different mRNA expression profiles were selected for WGCNA analysis (Fig. 3A, B, D and E). Firstly, selecting the appropriate soft threshold value according to R^2^ = 0.9, and finally choosing the soft threshold value β = 6 to establish the relationship matrix, then convert the relationship matrix into an adjacency matrix, and introduce power exponent weighting to construct a scale-free network, and finally in the adjacency matrix Based on the establishment of TOM matrix, calculate the degree of TOM difference between genes (distTOM), and then establish gene feature modules, obtaining a total of 24 modules. GO enrichment and KEGG pathway analysis were performed on the genes of different module eigengenes. According to GO function annotation and KEGG pathway enrichment analysis results, tumor-related modules are selected for subsequent analysis. In all modules, we found that there were more tumor-related enrichment results in the yellow module according to GO enrichment and KEGG pathway analysis. Finally, the yellow module is selected for further analysis. The yellow module has a total of 519 genes. The GO function annotation and KEGG enrichment analysis results (Supplementary Fig. A-D), and the correlation between the yellow module and the member genes (Fig. 3C).

### Construction of ceRNA network and topological analysis

**Fig. 4:**
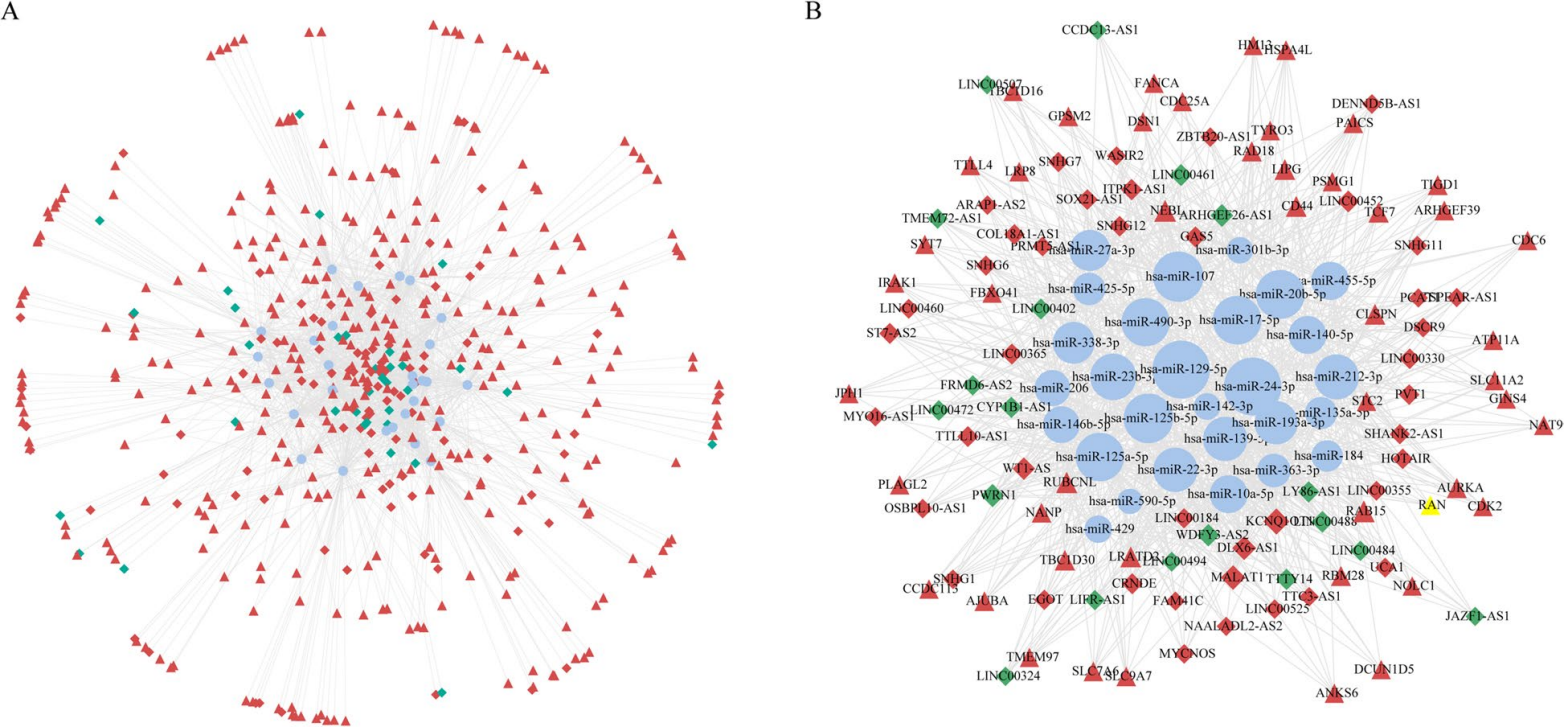
Construction of ceRNA regulation network using two relationship pairs. **A** is a ceRNA network diagram, and **B** is a sub-network composed of hub nodes with a degree greater than 5 in the ceRNA network. Diamond represents lncRNA, Triangle represents mRNA, Ellipse represents miRNA, red represents up-regulated genes, green represents down-regulated genes, and blue represents genes that are not differentially expressed (miRNAs)

The 519 mRNAs in the yellow module were predicted by miRNAs in the miRTarBase and miRWalk databases, and 21,725, 149,522 mRNA-miRNA relationship pairs were obtained, respectively. And 131,846 mRNA-miRNA relationship pairs were obtained through the intersection of the two shared miRNAs databases. At the same time, differential lncRNA was predicted by the miRNAs in the ENCORI and miRcode databases, and the prediction results were 15,788 and 5508 lncRNA-miRNA relationship pairs, respectively. The two were intersected by miRNA to obtain 1253 pairs of lncRNA-miRNA relationship. The mRNA-miRNA and lncRNA-miRNA are paired by miRNA, and then crossed with the colorectal cancer-related miRNA obtained from TCGA, and finally 31,900 pairs of lncRNA-miRNA-mRNA relationship pairs are obtained, and a ceRNA network is constructed based on this (Fig. 4A). Among them, 349 mRNAs were significantly up-regulated, 93 lncRNAs were significantly up-regulated and 34 lncRNAs were significantly down-regulated. At the same time, perform topological analysis on the ceRNA network, and select genes with a degree of greater than 5 to construct a sub-network (Fig. 4B), for the next step of analysis, which contains 61 lncRNAs, 48 mRNAs, and 28 miRNAs.

### Prognostic analysis of network nodes

The 61 lncRNAs, 48 mRNAs, and 28 miRNAs in the sub-network were grouped into Kaplan-Meier survival analysis with the smallest *p* value, and the Kaplan-Meier survival analysis of 14 lncRNAs, 20 mRNAs and 9 miRNAs was ultimately obtained.

**Fig. 5:**
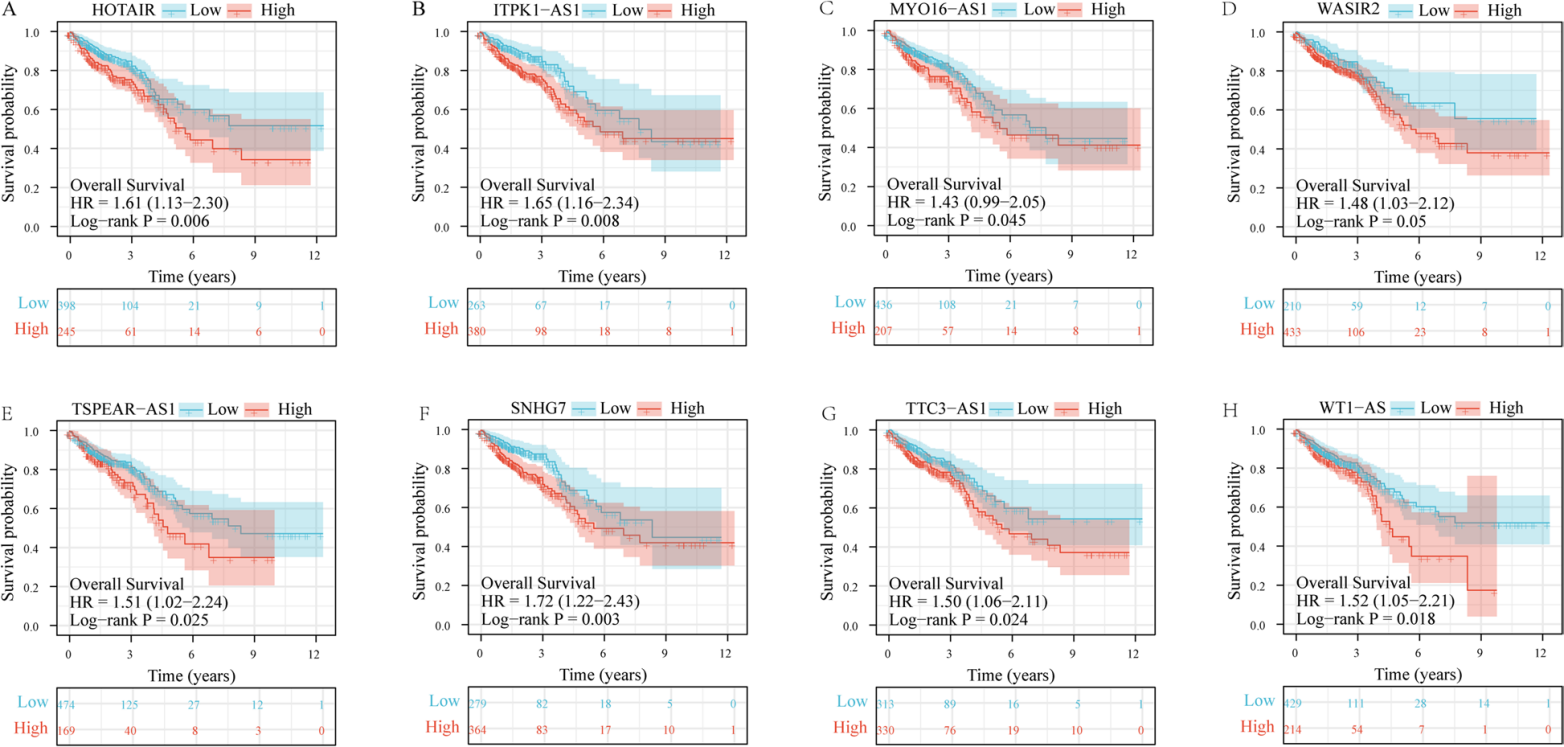
Significant lncRNAs for survival analysis in ceRNA sub-network. **A-H** is the survival analysis results of HOTAIR (*p* = 0.006), ITPK1-AS1 (*p* = 0.008), MYO16-AS1 (*p* = 0.045), WASIR2 (*p* = 0.05), TSPEAR-AS1 (*p* = 0.025), SNHG7 (*p* = 0.003), TTC3-AS1 (*p* = 0.024) and WT1-AS (*p* = 0.018) in the TCGA CRC data, respectively

**Fig. 6:**
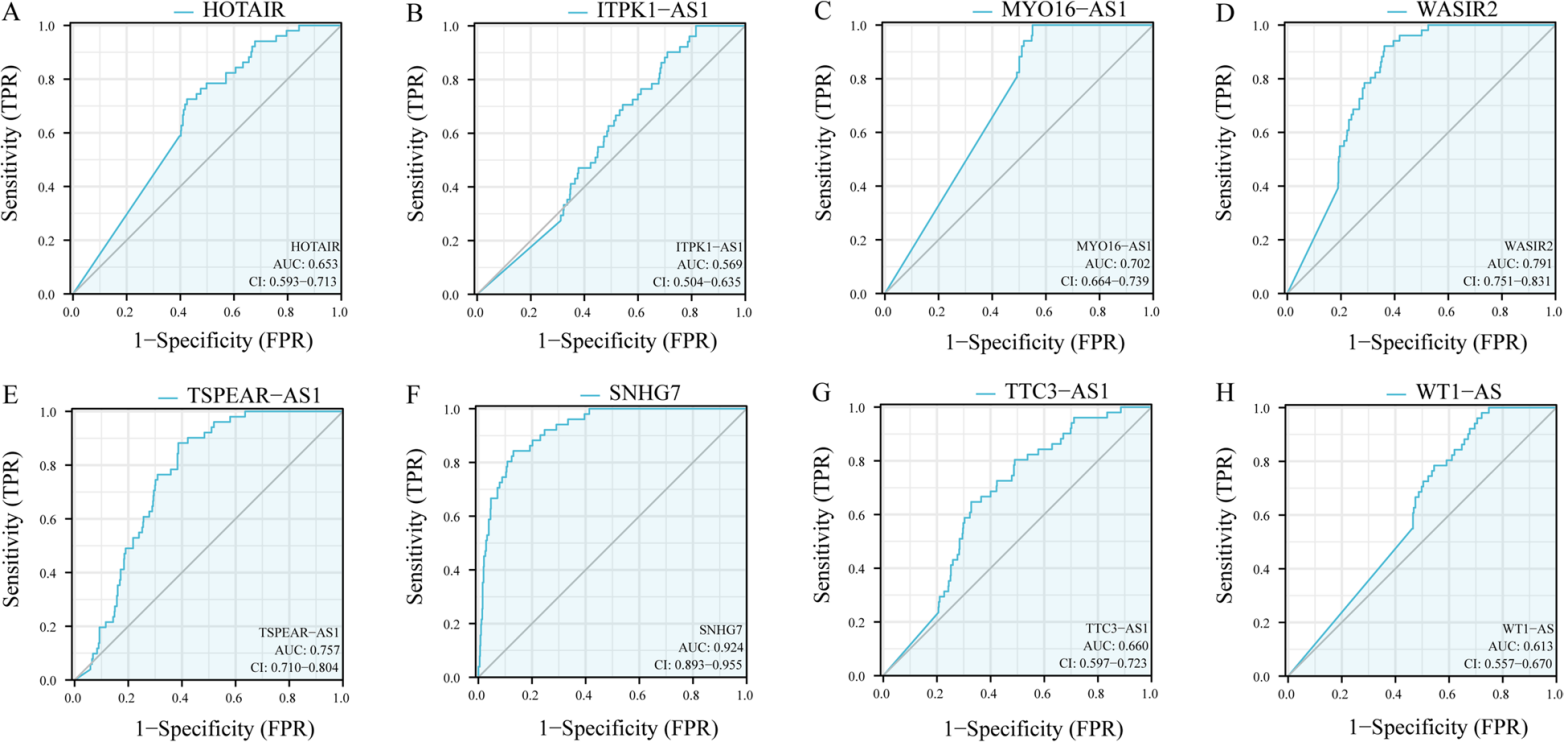
ROC curve analysis of lncRNAs in ceRNA sub-network. **A-H** are the ROC curves analysis results of HOTAIR (*p* = 0.005), ITPK1-AS1 (*p* = 0.012), MYO16-AS1 (*p* = 0.002), WASIR2 (*p* = 0.006), TSPEAR-AS1 (*p* = 0.043), SNHG7 (*p* = 0.001), TTC3-AS1 (*p* = 0.009) and WT1-AS (*p* = 0.025) in the TCGA CRC data, respectively

Among the 14 lncRNAs with significant differences in survival analysis, we discovered that patients with high expression of HOTAIR, ITPK1-AS1, MYO16-AS1, WASIR2, TSPEAR-AS1, SNHG7, TTC3-AS1 and WT1-AS had shorter survival time. At the same time, the HR of these 8 lncRNAs is more than 1, indicating that these 8 lncRNAs may be potential prognostic factors, as shown in Fig. 5A-H and Table 1. Then the ROC curve for these 8 lncRNAs was drawn and the AUC values were calculated. As shown in Fig. 6A-H, it can be found that the AUC values of the 8 lncRNAs are all greater than 0.5, indicating that it has good predictive value as a prognostic factor.

**Table 1:** Kaplan-Meier survival analysis significantly related RNA

	RNA	Log-rank P	HR	95%CI
lncRNA	HOTAIR	0.006	1.61	1.13-2.30
	ITPK1-AS1	0.008	1.65	1.16-2.34
	MYO16-AS1	0.045	1.43	0.99-2.05
	SNHG7	0.003	1.72	1.22-2.43
	TSPEAR-AS1	0.025	1.51	1.02-2.24
	TTC3-AS1	0.024	1.50	1.06-2.11
	WASIR2	0.05	1.48	1.03-2.12
	WT1-AS	0.018	1.52	1.05-2.21
mRNA	STC2	0.044	1.43	1.01-2.02
	TIGD1	0.007	1.60	1.12-2.28
miRNA	miR-17-5p	0.022	0.65	0.43-0.95
	miR-125b-5p	0.013	1.56	1.07-2.27
	miR-129-5p	0.032	0.66	0.46-0.95
	miR-193a-3p	0.004	1.65	1.15-2.35
	miR-206	0.018	0.61	0.42-0.88
	miR-212-3p	0.014	0.65	0.46-0.93
	miR-363-3p	0.008	1.71	1.19-2.46
	miR-425-5p	0.024	0.67	0.47-0.96
	miR-455-5p	0.041	0.69	0.47-1.01

**Fig. 7:**
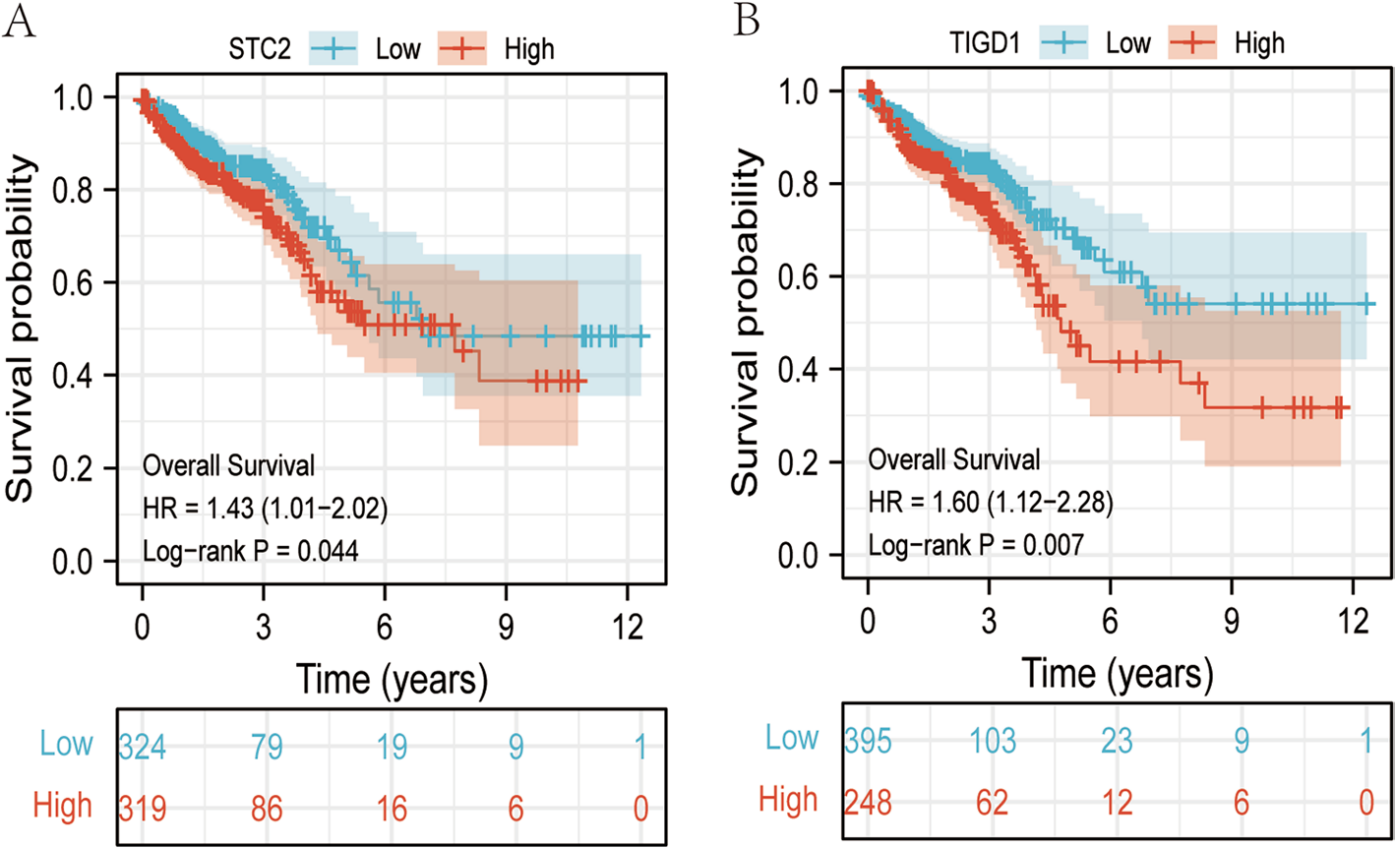
Significant mRNAs for survival analysis in ceRNA sub-network. **A-B** are the survival analysis results of STC2 (*p* = 0.044) and TIGD1 (*p* = 0.007) in the TCGA CRC data, respectively

**Fig. 8:**
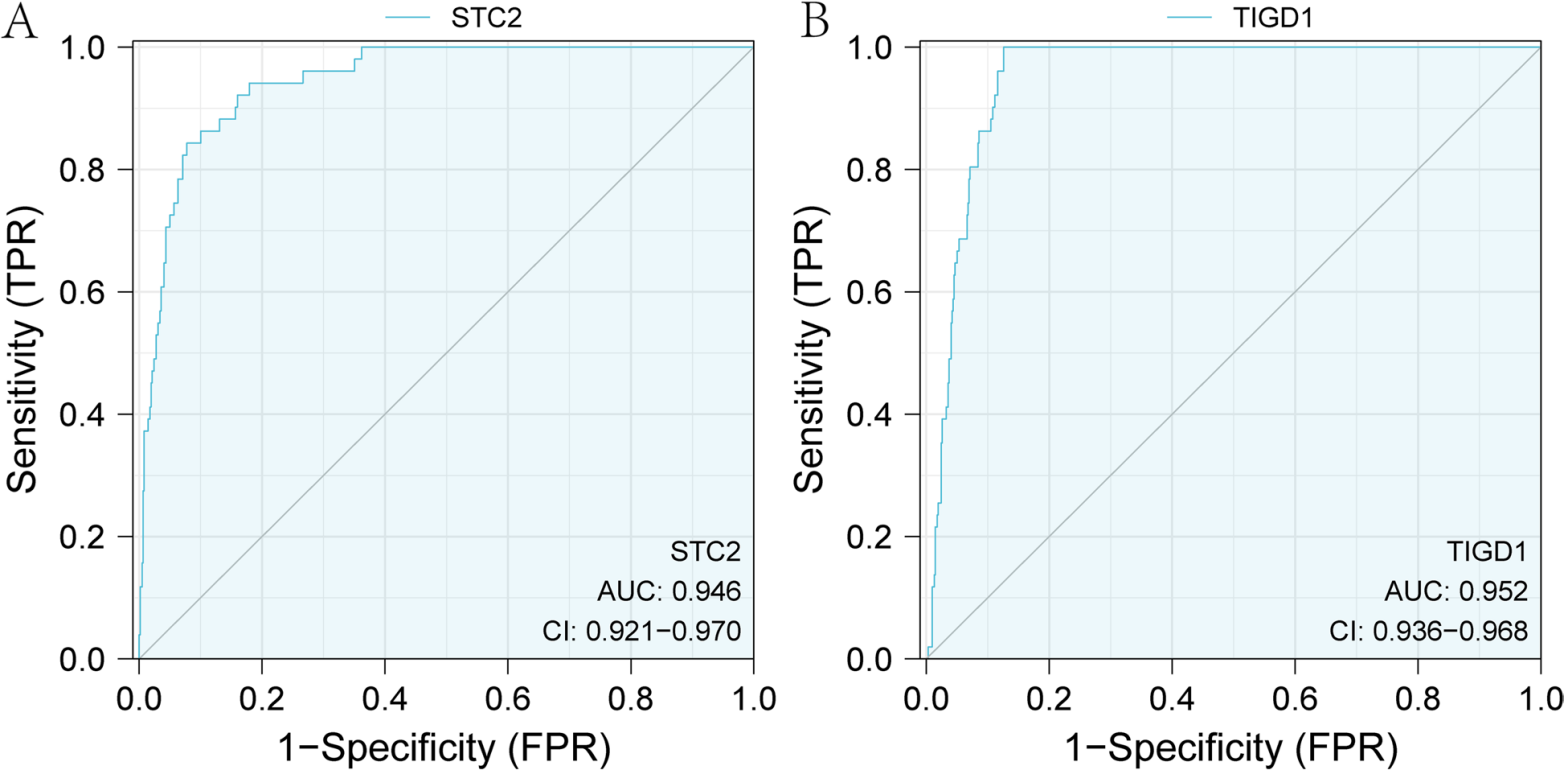
ROC curve analysis of mRNAs in ceRNA sub-network. **A-B** are the ROC analysis curve results of STC2(*p* = 0.035) and TIGD1(*p* = 0.027) in the TCGA CRC data, respectively

Among the 20 mRNAs with significant survival analysis, the survival time of the high expression group of STC2 and TIGD1 was shorter, and the HR was greater than 1 (Fig. 7, Table 1). It can be found that the survival analysis results of these two mRNAs indicate that STC2 and TIGD1 are both prognostic factors. Further ROC analysis indicates that STC2 and TIGD1 can discover that their AUC values are both greater than 0.9 (Fig. 8), demonstrating that they have good predictive value as prognostic factors.

**Fig. 9:**
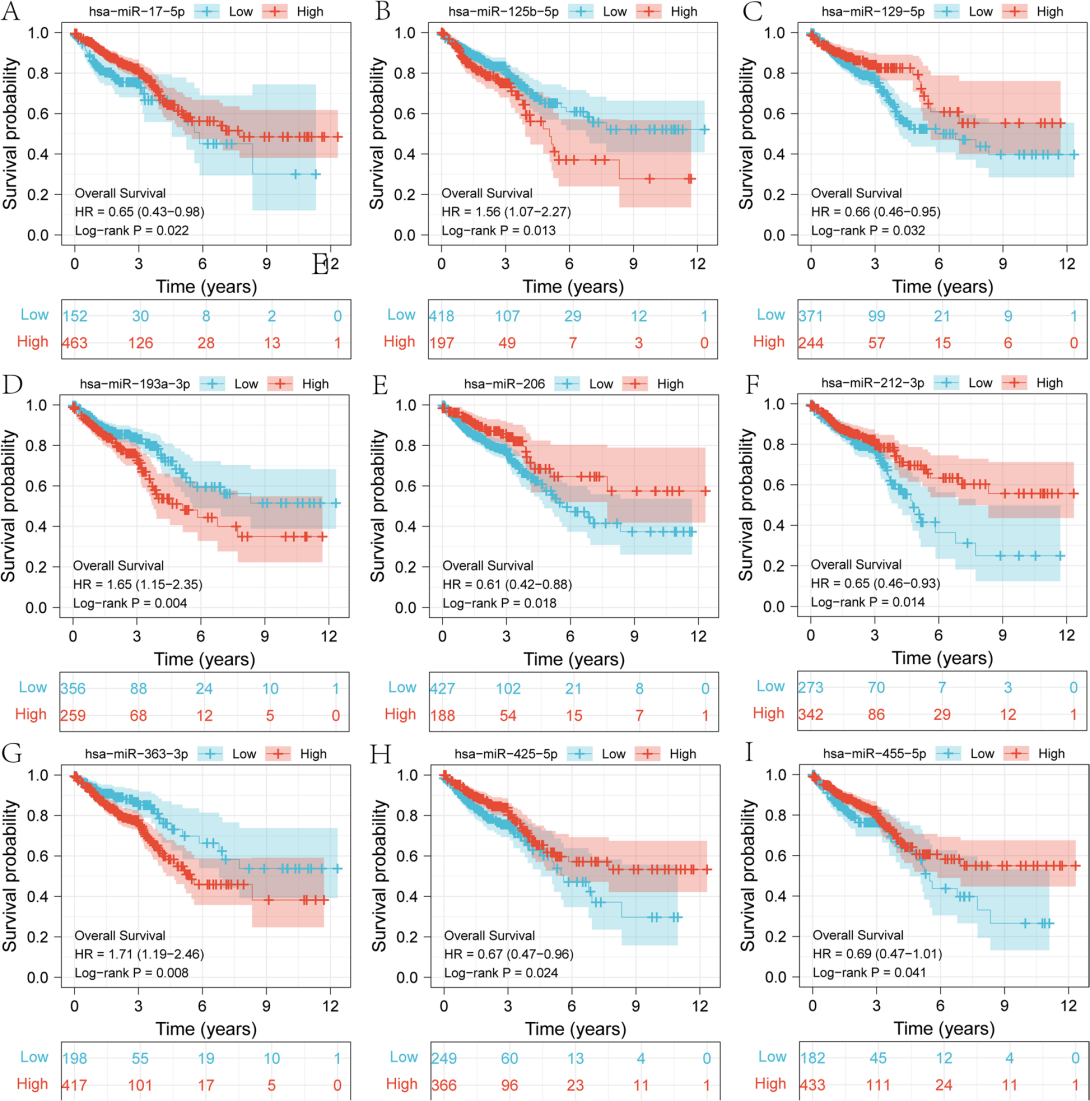
Significant miRNAs for survival analysis in ceRNA sub-network. **A-I** are the survival analysis results of miR-17-5p (*p* = 0.022), miR-125b-5p (0.013), miR-129-5p (*p* = 0.032), miR-193a-3p (*p* = 0.004), miR-206 (*p* = 0.018), miR-212-3p (*p* = 0.014), miR-363-3p (*p* = 0.008), miR-425-5p (*p* = 0.024) and miR-455-5p (*p* = 0.041) in the TCGA CRC data, respectively

**Fig. 10:**
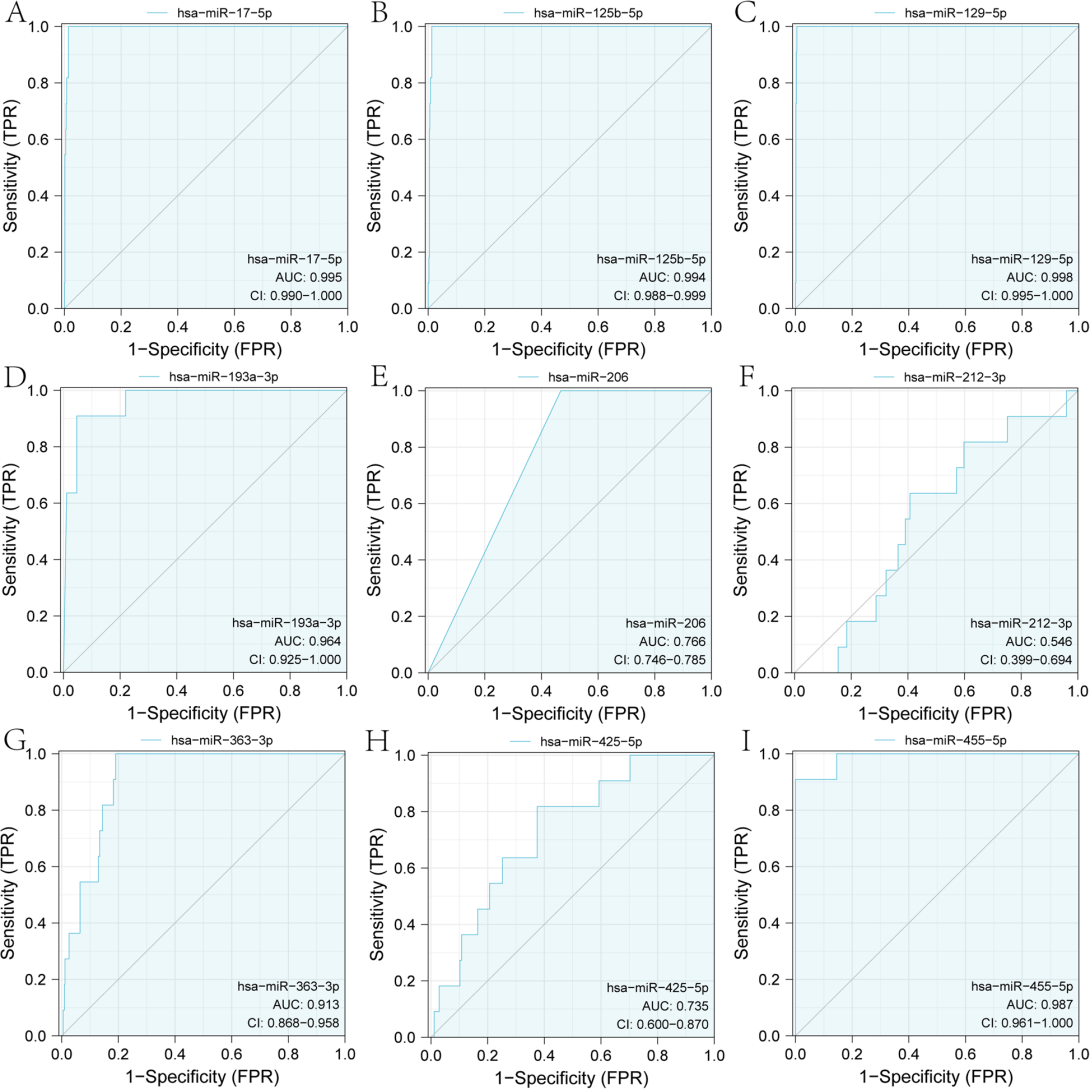
ROC curve analysis of miRNAs in ceRNA sub-network. **A-I** are the ROC analysis curve results of miR-17-5p (*p* = 0.00453), miR-125b-5p (*p* = 0.032), miR-129-5p (*p* = 0.001), miR-193a-3p (*p* = 0.005), miR-206 (*p* = 0.043), miR-212-3p (*p* = 0.002), miR-363-3p (*p* = 0.021), miR-425-5p (*p* = 0.019) and miR-455-5p (*p* = 0.004) in the TCGA CRC data, respectively

The survival analysis results of 9 significant miRNAs are shown in Fig. 9. Figure 9B, D and G results show that miRNA miR-125b-5p, miR-193a-3p and miR-363-3p survival analysis results, the high expression group. The survival time is shorter, and the survival time of the low expression group of miR-17-5p, miR-129-5p, miR-206, miR-212-3p, miR-425-5p and miR-455-5p is significantly longer short (Fig. 9A, C, E, F, H, I and Table 1). At the same time, we also performed ROC analysis on 9 miRNAs and found that their AUC values were all greater than 0.5, especially miRNA miR-17-5p, miR-125b-5p, miR-129-5p, miR-193a-3p and miR-455-5p, the AUC values are all greater than 0.95 (Fig. 10A-I).

Finally, we also calculated the correlation coefficient between the above RNAs, and the results are shown in Table 2.

**Table 2:** The correlation coefficient R between lncRNA, miRNA and mRNA

	LncRNA/mRNA	miRNA	R	*p*-value
lncRNA-miRNA	HOTAIR	miR-17-5p	0.12	0.068
	HOTAIR	miR-129-5p	0.29	0.046
	HOTAIR	miR-206	−0.44	0.025
	HOTAIR	miR-193a-3p	0.23	0.6
	ITPK1-AS1	miR-212-3p	−0.11	0.016
	ITPK1-AS1	miR-129-5p	−0.10	0.025
	ITPK1-AS1	miR-17-5p	0.04	0.94
	ITPK1-AS1	miR-455-5p	0.02	0.75
	MYO16-AS1	miR-125b-5p	0.56	0.00049
	MYO16-AS1	miR-129-5p	0.62	0.028
	MYO16-AS1	miR-425-5p	0.28	< 0.0001
	SNHG7	miR-425-5p	0.33	< 0.0001
	SNHG7	miR-193a-3p	0.62	< 0.0001
	TSPEAR-AS1	miR-193a-3p	0.42	< 0.0001
	TSPEAR-AS1	miR-212-3p	0.21	< 0.0001
	TTC3-AS1	miR-193a-3p	0.32	< 0.0001
	TTC3-AS1	miR-363-3p	0.26	< 0.0001
	WASIR2	miR-129-5p	0.20	< 0.0001
	WASIR2	miR-455-5p	0.24	< 0.0001
	WASIR2	miR-193a-3p	0.19	0.00027
	WT1-AS	miR-17-5p	−0.57	0.0085
	WT1-AS	miR-206	−0.49	0.043
	WT1-AS	miR-129-5p	0.07	0.012
	WT1-AS	miR-125b-5p	0.22	< 0.0001
	WT1-AS	miR-193a-3p	0.64	0.0018
mRNA-miRNA	STC2	miR-129-5p	0.17	0.00016
	STC2	miR-455-5p	0.35	< 0.0001
	STC2	miR-125b-5p	0.51	< 0.0001
	TIGD1	miR-129-5p	0.63	< 0.0001
	TIGD1	miR-455-5p	0.42	< 0.0001
	TIGD1	miR-212-3p	0.40	< 0.0001

### Analysis of expression differences of network nodes

At the same time, the survival analysis based on the sub-network nodes is meaningful, and we also investigated whether there is a difference in the expression of the nodes in the tumor and normal tissues.

**Fig. 11:**
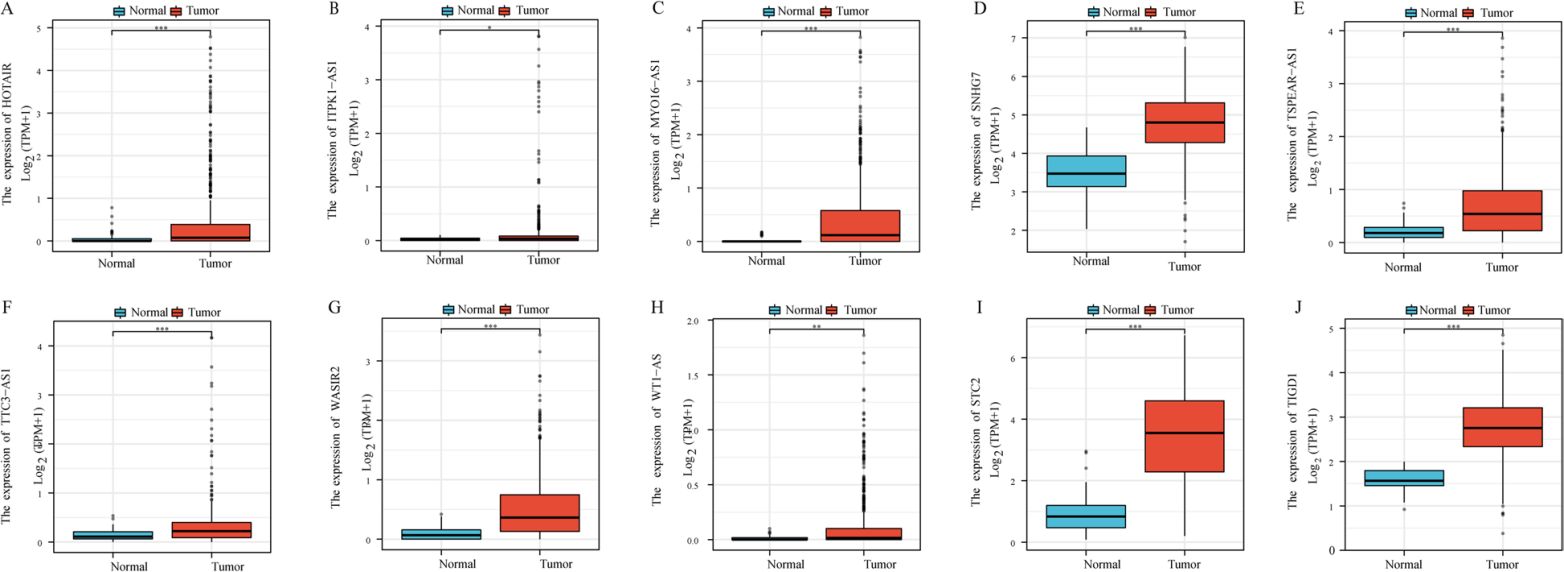
Differential expression of lncRNA and mRNA in TCGA CRC data. **A-J** are the expression differences results of HOTAIR, ITPK1-AS1, MYO16-AS1, WASIR2, TSPEAR-AS1, SNHG7, TTC3-AS1, WT1-AS, STC2 and TIGD1, respectively. One asterisk represents *p* < 0.05, two asterisks represent *p* < 0.01, and three asterisks represent *p* < 0.001

**Fig. 12:**
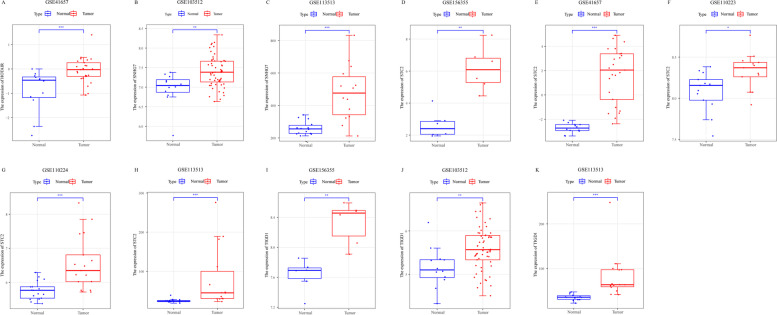
Differential expression of lncRNA and mRNA in GEO database. **A** shows the differential expression analysis of HOTAIR in GSE41657, **B** and **C** shows the differential expression analysis of SNHG7 in GSE103512 and GSE113513, respectively. **A** and **D-H** shows the expression analysis of STC2 in GSE156355, GSE41657, GSE110223, GSE110224, and GSE113513, respectively. **I-K** is the difference analysis of TIGD1 expression in GSE156355, GSE103512 and GSE113513, respectively. One asterisk represents *p* < 0.05, two asterisks represent *p* < 0.01, and three asterisks represent *p* < 0.001

Through the analysis of the expression levels of the above 8 lncRNAs in TCGA, it is discovered that they are all highly expressed in the TCGA CRC data (Fig. 11A-H). What is interesting is that mRNA STC2 and TIGD1 likewise exhibit high expression (Fig. 11I-J). To verify whether the above gene expression differences are universal, we used GEO data to verify their expression levels. The analysis results of some lncRNA and mRNA expression differences are displayed in Fig. 12. We can discover that these lncRNA or mRNA also appear to be high expression in GEO data.

**Fig. 13:**
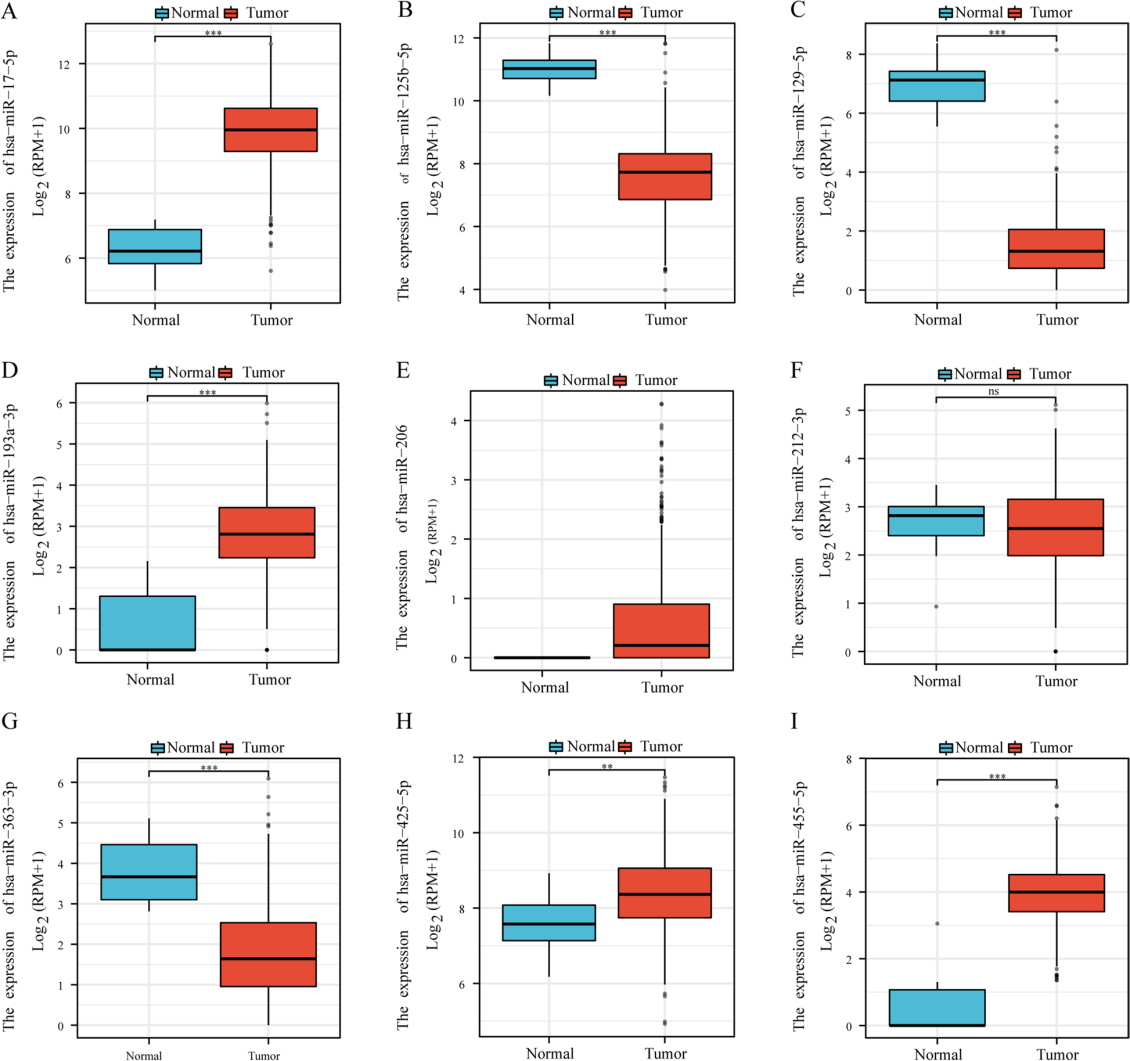
Differential expression of miRNA in TCGA CRC data. **A-I** are the differential expression results of miR-17-5p, miR-125b-5p, miR-129-5p, miR-193a-3p, miR-206, miR-212-3p, miR-363-3p, miR-425-5p and miR-455-5p, respectively. One asterisk represents *p* < 0.05, two asterisks represent *p* < 0.01, and three asterisks represent *p* < 0.001

Finally, we also investigated the expression of 9 miRNAs and found that miR125b-5p, miR-129-5p and miR-363-3p are low in tumor tissues, while miR-17-5p and miR-193a- 3p, miR-206, miR-212-3p, miR-425-5p and miR-455-5p are highly expressed in tumor tissues (Fig. 13).

## Discussion

In 2011, Salmena et al. proposed the establishment of a new RNA regulatory network hypothesis, that is, mRNA and lncRNA regulate each other through miRNA as a bridge [15]. The basis for the two to regulate each other is the existence of miRNA reflection files (MREs) [15]. We already know that miRNA has a regulatory effect on mRNA, and lncRNA antagonizes this regulatory effect by competing with miRNA, which forms a regulatory network. This competitive endogenous RNA (ceRNA) communication forms a large-scale regulatory network spanning the transcriptome, which greatly expands the functional genetic information in the human genome and plays a role in pathological conditions, including cancer played an important role [21]. Formerly, the Cancer Genome Atlas (TCGA) analyzed the relationship between gene expression data of colon cancer and the pathological stage [22], and identified potential prognostic miRNA biomarkers for predicting the overall survival of colon cancer [23]. Considering that the weighted gene co-expression network (WGCNA) has good clustering efficiency for genes with the same expression pattern, and in previous ceRNA network studies, researchers did not incorporate it with WGCNA when constructing ceRNA network. In this study, we combined the ceRNA network and WGCNA for the first time to discover more and more reliable tumor-related genes. The purpose for finding new lncRNA-miRNA, miRNA-mRNA or lncRNA-miRNA-mRNA axis, which can regulate the development, metastasis, proliferation and invasion of CRC, is to provide new ideas for studying the molecular mechanisms of the initiation and progression of CRC, and at the same time provide new targets for the treatment of CRC.

Studies have found that lncRNA is related to a variety of biological regulatory functions such as epigenome, transcription or posttranscriptional levels, and cancer pathogenicity [24–26]. In this study, we analyzed and screened out 14 lncRNAs with meaningful survival analysis, including 3 down-regulated lncRNAs and 11 up-regulated lncRNAs, and further screened out 8 lncRNAs with significant high expression in colorectal cancer. Namely HOTAIR, ITPK1-AS1, MYO16-AS1, WASIR2, TSPEAR-AS1, SNHG7, TTC3-AS1 and WT1-AS. Among these lncRNAs, a portion of lncRNA has been confirmed by previous molecular experiments, such as p21-mediated down-regulation of HOTAIR to inhibit the proliferation, invasion and metastasis of CRC cells [27]. Furthermore, lncRNA SNHG7 sponging miR216b promotes liver metastasis of CRC by up-regulating GALNT1 [28], while down-regulation of SNHG7 can inhibit the phenotype of malignant bladder cancer [29]. The decreased expression of another lncRNA WT1-AS can promote the proliferation and invasion of gastric cancer cells [30]. The above results demonstrate the reliability of our research to a certain extent. Hence, the lncRNAs discovered in this study may be used as biomarkers and have potential applications in the diagnosis, progression and therapy of CRC.

Previous studies have indicated that miRNAs post-transcriptionally regulate gene expression in multifarious cancer-related signaling pathways and processes [31], and miRNAs can reduce mRNA stability or inhibit translation by binding to MRE. By analyzing TCGA CRC data, we have summarized 8 miRNAs. Some of the above miRNAs have been reported: for example, in CRC, miR-17-5p regulates EMT through targeted vitamins [32] and predicts the pathological staging and grading of CRC [33]. At the same time, research reports have identified that miR-206 regulates the resistance of intestinal cancer cells to 5-FU by targeting BCL2 [34]. In addition, miR-125b-5p and miR-17-5p can predict liver metastasis and chemotherapy response in advanced CRC [35]. At present, the understanding of other miRNAs discovered in this study is insufficient, and molecular studies are required to verify this. These colorectal cancer-specific miRNAs may at some point become specific potential biomarkers in the diagnosis and progression of CRC.

The ceRNA network we constructed is primarily via lncRNA-miRNA and miRNA-mRNA relationship pairs, as shown in Tables 3 and 4. In these analysis results, some regulatory networks have been reported, such as the HOTAIR sponge miR-17-5p, which plays a tumor-promoting role in cervical cancer [36]. At the same time, the HOTAIR / miR-206 axis occupies an important position in the occurrence and development of various tumors. For example, the HOTAIR sponge miR-206 regulates STC2 and affects the biological functions of head and neck squamous cell cancer cells [37]. HOTAIR uses miR-The 206/TBX3 axis maintains the stemness of ovarian cancer stem cells [38] and HOTAIR regulates the proliferation of breast cancer cells through the miR-206-mediated BCL-W signaling pathway [39]. In addition, it has been reported that propofol inhibits cervical cancer progression by regulating the HOTAIR/miR-129-5p/RPL14 axis [40] and HOTAIR promotes breast cancer progression by regulating the miR-129-5p/FZD7 axis [41]. Based on previous reports and our analysis results, it has been discovered that HOTAIR plays an important role in the occurrence and development of various cancers. We can assume that the HOTAIR-miR-17-5p, HOTAIR-miR-206, or miR-129-5p axis also play an important role in the occurrence and development of CRC, especially the HOTAIR/miR-206/STC2 axis in CRC. It plays the same role in cancer, just like the HOTAIR / miR-206 / STC2 axis in head and neck squamous cell carcinoma.

**Table 3:** lncRNA-miRNA relationship pairs

lncRNA	miRNA
HOTAIR	hsa-miR-17-5p
	hsa-miR-129-5p
	hsa-miR-206
	hsa-miR-193a-3p
ITPK1-AS1	hsa-miR-212-3p
	hsa-miR-129-5p
	hsa-miR-17-5p
	hsa-miR-455-5p
MYO16-AS1	hsa-miR-125b-5p
	hsa-miR-129-5p
	hsa-miR-425-5p
SNHG7	hsa-miR-425-5p
	hsa-miR-193a-3p
TSPEAR-AS1	hsa-miR-193a-3p
	hsa-miR-212-3p
TTC3-AS1	hsa-miR-193a-3p
	hsa-miR-363-3p
WASIR2	hsa-miR-129-5p
	hsa-miR-455-5p
	hsa-miR-193a-3p
WT1-AS	hsa-miR-17-5p
	hsa-miR-206
	hsa-miR-129-5p
	hsa-miR-125b-5p
	hsa-miR-193a-3p

**Table 4:** mRNA-miRNA relationship pairs

mRNA	miRNA
STC2	hsa-miR-129-5p
	hsa-miR-455-5p
	hsa-miR-125b-5p
TIGD1	hsa-miR-129-5p
	hsa-miR-455-5p
	hsa-miR-212-3p

In this study, there are currently two main limitations. The first is that clinical information such as TNM, sex, age, tumor staging and histology have not been analyzed. This may be a potential limitation that requires further research to clarify; and the other is that we just based on the CRC data in TCGA, the theoretical analysis is carried out, and the specific molecular mechanism needs to be confirmed by experiments. In the future, molecular biology methods such as qPCR, luciferase reporter system and co-immunoprecipitation will help verify our findings and clarify the molecular mechanism of the ceRNA network.

## Conclusion

We identified 8 lncRNAs, 2 mRNAs and 9 miRNAs in the ceRNA network, which have prognostic significance for patients with CRC. Their interaction constitutes the lncRNA-miRNA-mRNA axis. The prognostic significance of these RNA molecules in CRC patients may indicate that these lncRNA-miRNA-mRNA axes play an important role in the occurrence and development of CRC. ​ The lncRNA-miRNA-mRNA axes may potentially uncover new molecular mechanisms of CRC occurrence and development and provide an important blueprint for the treatment and prognosis evaluation of CRC patients.

## Supplementary Information

**Additional file 1: Supplementary Fig.** GO enrichment and KEGG pathway analysis. A-D are the GO function annotation and KEGG pathway enrichment analysis figure in the yellow module, respectively.

## Data Availability

The data of this study are from GEO (https://www.ncbi.nlm.nih.gov/geo/) and TCGA database (https://portal.gdc.cancer.gov/).
