# Supplementary material for: Construction of CeRNA regulatory network based on WGCNA reveals diagnosis biomarkers for colorectal cancer
Source: BMC Cancer. 2022 Sep 17;22:991. doi: 10.1186/s12885-022-10054-z (PMC9482270; doi:10.1186/s12885-022-10054-z)
Supplement: Supplementary file 1 — Additional file 1: Supplementary Fig. GO enrichment and KEGG pathway analysis. A-D are the GO function annotation and KEGG pathway enrichment analysis figure in the yellow module, respectively. [file 12885_2022_10054_MOESM1_ESM.pdf]

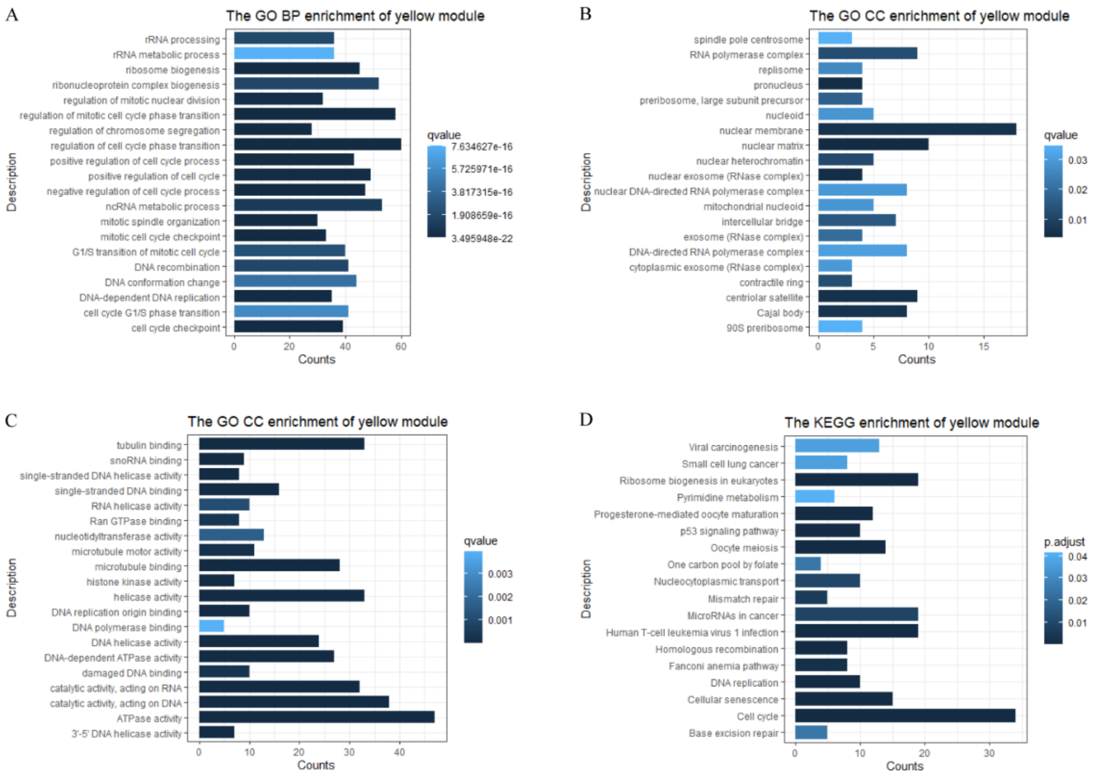

Supplementary Figure. GO enrichment and KEGG pathway analysis. The Figure A-D are the GO function annotation and KEGG pathway enrichment analysis figure in the yellow module, respectively.
